# Occult Perirectal Abscess Causing Acute Urinary Retention

**DOI:** 10.7759/cureus.12461

**Published:** 2021-01-03

**Authors:** Steve W Updike, Zachary Sletten

**Affiliations:** 1 Emergency Medicine, San Antonio Military Medical Center, San Antonio, USA

**Keywords:** perirectal abscess, urinary retention, aur

## Abstract

Acute urinary retention (AUR) is a common symptom evaluated in the emergency department. It is generally due to an obstructive process such as benign prostatic hyperplasia (BPH) and can be treated simply with an indwelling foley catheter and urological follow up. Perirectal abscess is a relatively rare cause of urinary retention with no documented prevalence but when present is almost universally accompanied by perirectal pain. We present a 53-year-old male with a four-day history of urinary retention without perirectal pain or additional symptoms, who was found to have a perirectal abscess on digital rectal exam (DRE) and confirmed on computed tomography (CT) imaging.

## Introduction

Acute urinary retention (AUR) is relatively common, affecting men more than women, and it is estimated that 10% of men will experience AUR by age 70 and 33% by age 80 [1], whereas women account for only 3 out of every 100,000 cases annually [2]. Although the most common etiology is benign prostatic hyperplasia (BPH) [3], the differential is broad and includes obstructive, infectious (e.g. UTI), traumatic (e.g. GU or spinal cord injuries), pharmacologic (e.g. anticholinergic, sympathomimetic, etc.), and neurologic (e.g. diabetic, both upper and lower motor neuron) causes [1,3]. A genital and rectal examination should be performed as a part of the initial workup of AUR looking for evidence of etiologies such as prostatitis in men and uterine prolapse in women along with a detailed neurologic exam. An uncommon cause of AUR, perirectal abscesses can cause retention by mechanical obstruction of the urinary outflow tract as well as by surrounding inflammation. Patients with perirectal abscesses frequently have associated perirectal pain in addition to concerning digital rectal exam (DRE) [4]. Without an appropriately focused history and physical exam, this process can be easily missed with resultant complications including bacteremia, fistula formation, fecal incontinence, urinary incontinence, and chronic pain [5]. The authors present a case of a perirectal abscess causing AUR.

## Case presentation

A 53-year-old male presented to the emergency department (ED) with progressive urinary retention for four days and stated he was unable to void for the last 12 hours. He denied any abdominal pain but admitted to fullness to his lower abdomen. His past medical history was unremarkable and specifically, he had no history of BPH, urinary obstruction, or recent instrumentation. He denied recent use of narcotics, nonsteroidal anti-inflammatory drugs (NSAIDs), antihistamines such as diphenhydramine, cough medicines such as pseudoephedrine, or other new medications. On review of systems, the patient denied fever, chills, rectal pain, weakness or numbness, back pain, dysuria, or frequency prior to symptom onset.

On exam, he was afebrile with normal vitals. His abdominal exam was notable for increased pressure to the suprapubic area. External genital exam, including perineum, was unremarkable. The neurologic exam on the patient was unremarkable, including intact lower extremity sensation to light touch, intact muscle strength to all lower extremity muscle groups, and intact deep tendon reflexes to the bilateral patellar and Achilles tendons.

Point of care ultrasound demonstrated significant urinary retention that estimated the volume to be greater than 975 cc of urine. A foley catheter was placed with relief of pressure and return of 2.5 L of straw-colored urine. Following foley placement, a DRE was performed. No overlying skin changes or hemorrhoids were noted. On internal exam, he had exquisite tenderness in the anterior direction with no obvious induration or fluctuance. The degree of internal tenderness noted on the exam raised concern for an infectious process such as a perirectal abscess. A urinalysis, basic metabolic panel (BMP), and complete blood count (CBC) were obtained. There were no signs of infection on urinalysis. No renal dysfunction was seen on the BMP and there was a mild leukocytosis at 12,000 cells/mm^3 and left shift of neutrophils on the CBC.

**Figure 1:**
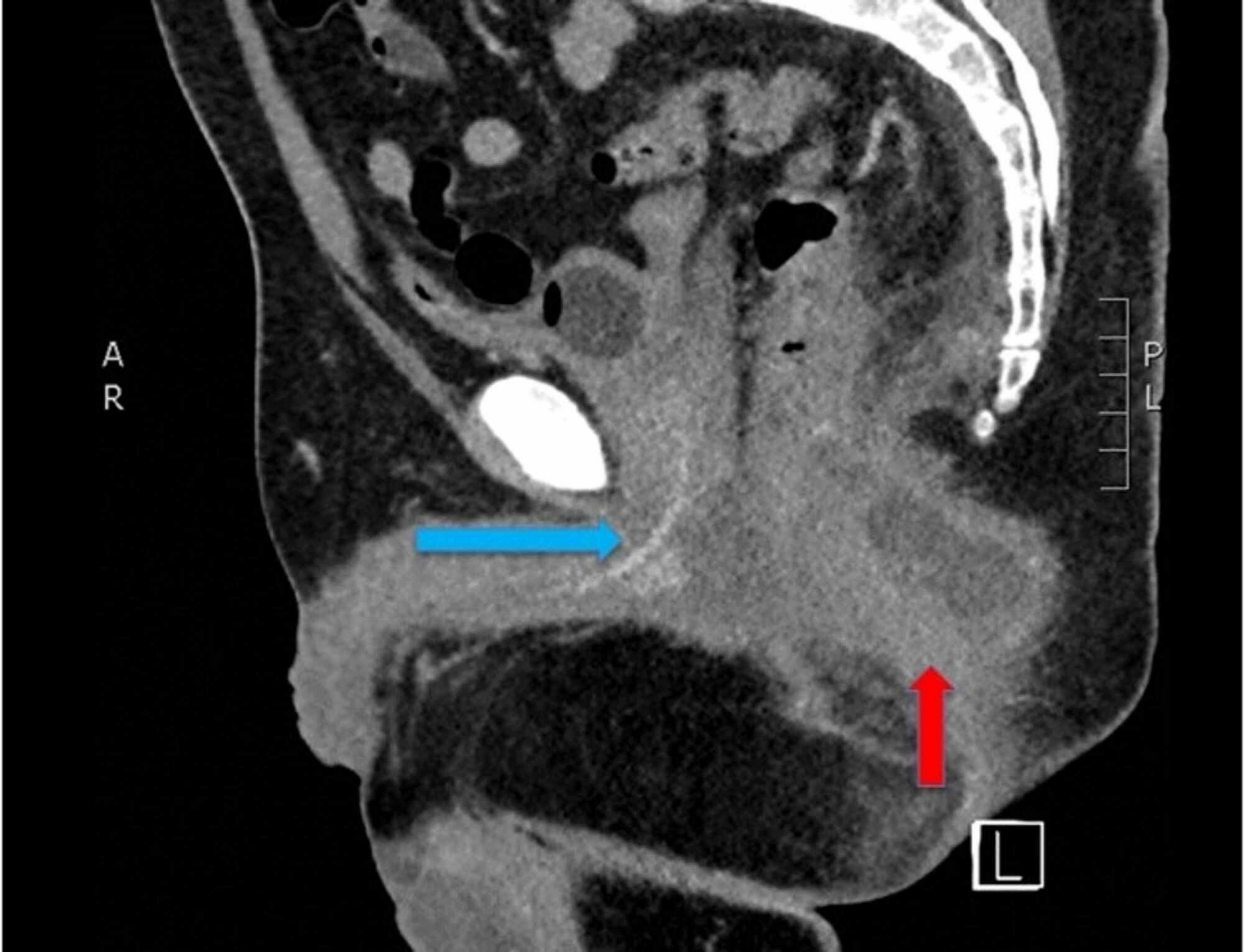
Sagittal Slice CT Pelvis with IV Contrast Ring enhancing lesion posterior to rectum (red arrow). And anterior is foley catheter (blue arrow).

**Figure 2:**
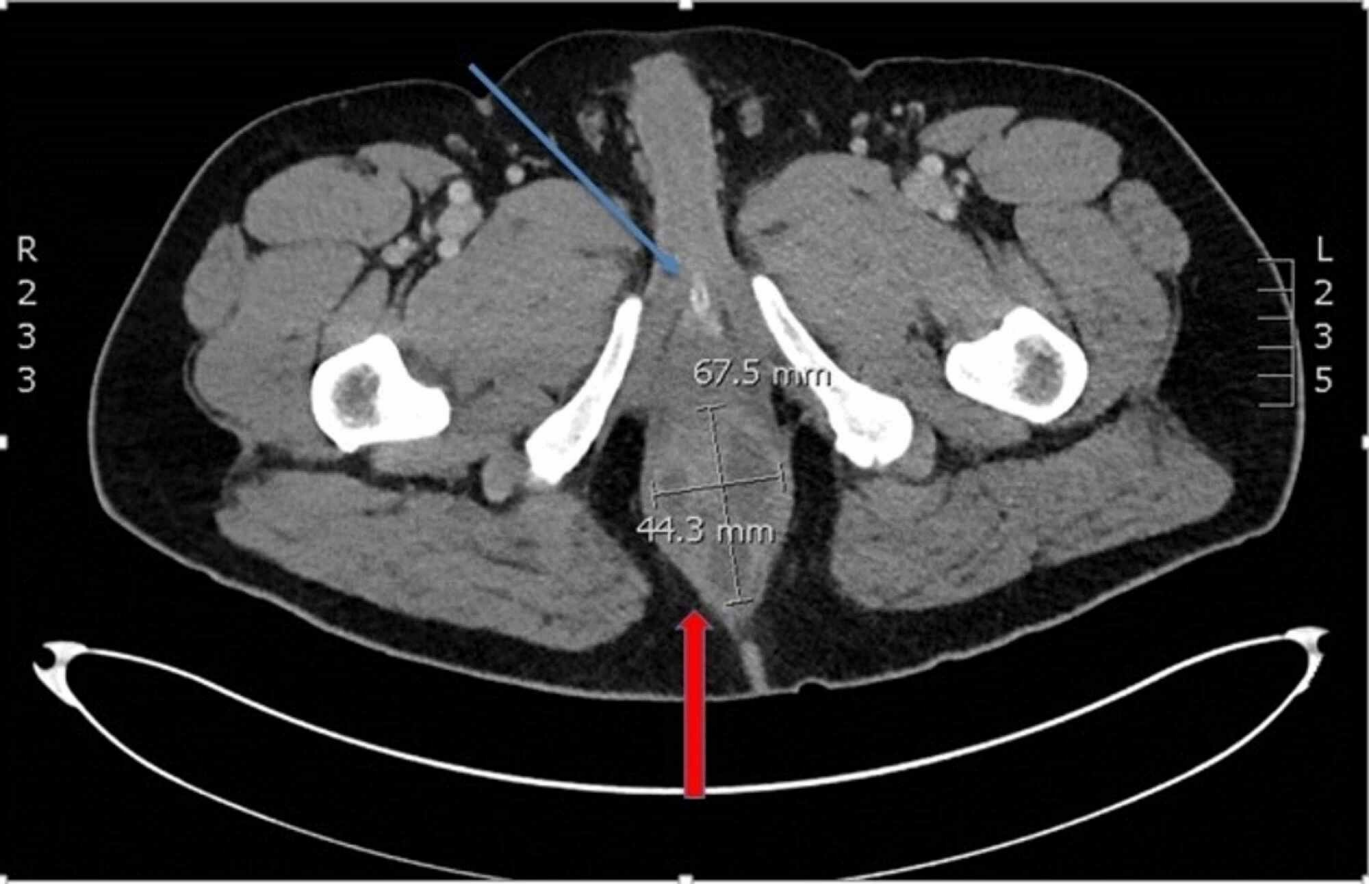
Transverse Slice of CT Pelvis with IV Contrast Ring enhancing lesion posterior to rectum (red arrow) and foley catheter (blue arrow).

A computed tomography (CT) scan of the pelvis using intravenous (IV) contrast was performed, which demonstrated a 7.6 x 4.4 x 6.8 cm heterogeneous mass with multiloculated, hypoattenuating fluid components, and rim enhancement concerning for perirectal abscess (Figures 1-2).

The patient was started on IV piperacillin/tazobactam 3.375 grams and general surgery was consulted. The patient was taken to the operating room for definitive treatment with incision and drainage. Surgical exploration revealed an ischio-rectal abscess, more specifically “horseshoe” abscess. The patient was discharged the next day and followed up one week later with relief of symptoms.

## Discussion

AUR is a common complaint, with a broad differential. Etiologies can be categorized as obstructive, infectious, traumatic, pharmacologic, and neurologic. The most common etiology is obstructive secondary to BPH in men and in women from bladder masses, surgical complications, and pelvic organ prolapse [6]. A focused review of systems and past medical history combined with a focused exam may elucidate the cause of AUR. Evaluating clinicians should ask about the use of medications known to cause AUR such as cough medicine, NSAIDs, or opiates [1]. Of note, a myriad of medications can trigger urinary retention, mostly due to either sympathomimetic or anticholinergic effects on the smooth muscles of the urinary system.

Dysuria, frequency, or fever may suggest urinary tract infection or prostatitis whereas an insidious onset of difficulty voiding may suggest BPH. Recent sensation loss or muscle weakness with or without back pain is evidence of neurologic etiology such as cauda equina, spinal cord compression, or multiple sclerosis. The DRE may show decreased tone, increasing concern for spinal cord pathology such as cauda equina. A detailed neurologic exam of the lower extremity is useful, especially in identifying other neurologic causes including Guillain-Barre syndrome, diabetic neuropathy, and multiple sclerosis [1,6]. Examination to see if the penis has foreskin findings concerning for paraphimosis or phimosis to causes an obstructive process [1]. Significant rectal pain and tenderness on DRE should raise concern for perirectal abscess. 

Ano-rectal abscesses are rather uncommon with an incidence of 1 in 10,000, affecting males three times more often than females [5]. Cases per year estimated to be around 68 to 96,000 [7] and patients are typically in their forties [8]. The majority of abscesses are caused by an infected anal crypt gland located at the level of the dentate line, however, up to 10% are thought to result from another cause such as Crohn's disease, human immunodeficiency virus (HIV), trauma, or radiation. Interestingly, there does not appear to be an association with diabetes [5}. Infection occurs as the gland becomes inflamed or obstructed, leading to stasis and ultimately bacterial overgrowth [7,8]. Infection of these anal glands can track in any direction into different anatomic spaces. A downward extension results in a perianal abscess. The infection can track laterally across the external sphincter into the ischiorectal fossa (ischiorectal fossa abscess). Less common infection can extend superiorly up the intersphincteric groove to the supralevator space or in the submucosal plane [8]. Extension to the perianal area resulting in a perianal abscess is by far the most common in 70% of cases, while ischiorectal extension is second most common at 20% [5]. While simple perianal abscesses can be incised and drained at bedside, peri-rectal abscess such as ischiorectal abscesses require intravenous antibiotics [4] and surgical drainage [5,8]. Common bacteria isolated from the perirectal cultures were found to be anaerobic and gram-negative bacteria including Bacteroides fragilis, Peptostreptococcus, Prevotella, Fusobacterium, Porphyromonas, Clostridium species, and Escherichia coli [9].

Ano-rectal abscesses can cause obstruction directly through compression of the urethra or secondarily through local inflammation of surrounding tissue. A retrospective study of 92 patients diagnosed with a rectal abscess [4] showed that of patients, 4% presented with urinary symptoms such as retention or frequency while rectal pain was the most common symptom and was reported in 98.9% of cases. An abnormal DRE concerning for abscess was noted in 94% of these patients [4]. Additionally, other case reports have shown that patients often experience fevers [10]. If there is concern for a perirectal abscess, a CT pelvis with IV contrast can confirm the diagnosis. Other imaging modalities that can be useful include ultrasound and magnetic resonance imaging (MRI) [7,5].

In the above case, the patient presented without rectal pain, fever, or any predisposing conditions but was ultimately discovered to have an abscess after a concerning DRE. To this authors’ knowledge, this is the first written case report of an anorectal abscess presenting as AUR without any pain or systemic symptoms. This serves to reinforce the importance of a focused but thorough evaluation to include a DRE of patients presenting with unexplained AUR, even in the absence of rectal pain. The patient went on to be diagnosed with a horseshoe abscess, which is a specific type of ischiorectal abscess. This abscess is caused by an infected anal gland located midline posterior to the anal canal [7,8]. The superior anococcygeal ligament blocks the direct downward expression of an abscess preventing a perianal abscess. This results in extension bilaterally into the ischiorectal fossa, causing a horseshoe shape [7,8]. Treatment requires surgical unroofing and drainage, like other ischiorectal abscesses.

Similar case reports showed correlations of rectal pain and urinary retention, which found ischiorectal abscess to be the culprit, one of which developed severe soft tissue infection and sepsis due to delayed treatment [11]. This demonstrates early surgical consultation and drainage for source control is imperative. Antibiotic therapy alone is insufficient for the treatment of ischiorectal abscess due to occluded and necrotic blood vessels with subsequently poor antibiotic penetration into the abscess cavity [7]. Complications from ischiorectal abscess include bacteremia, sepsis, fistula formation, and fecal incontinence. Recurrence rates and long term morbidity are high and up to 30% of patients may have chronic rectal pain after the resolution of the abscess [5].

## Conclusions

AUR is relatively common, particularly for older men and has a broad differential. A focused yet thorough history and physical exam can be helpful in arriving at the correct diagnosis and avoiding pitfalls. Areas to focus on the physical exam should be the abdominal, genitourinary, neurological, and rectal exam. Early identification of perirectal abscess in AUR can mitigate morbidity and mortality. Classical historical clues, to include rectal pain in the case of perirectal abscess, may be absent. In the case presented above, the DRE was essential in diagnosing a perirectal abscess that presented without common symptoms such as rectal pain, fever or malaise and without any predisposing past medical history. Evaluation in the ED with inclusion of a DRE for unexplained AUR should be performed to evaluate for perirectal abscess.
